# Magnitude of first line antiretroviral therapy treatment failure and associated factors among adult patients on ART in South West Shoa, Central Ethiopia

**DOI:** 10.1371/journal.pone.0241768

**Published:** 2020-11-11

**Authors:** Diriba Mulisa, Tadesse Tolossa, Bizuneh Wakuma, Werku Etafa, Girma Yadesa

**Affiliations:** 1 School of Nursing and Midwifery, Institutes of Health Sciences, Wollega University, Nekemte, Ethiopia; 2 Department of Public Health, Institutes of Health Sciences, Wollega University, Nekemte, Ethiopia; 3 Department of Pediatric Nursing, Institutes of Health Sciences, Wollega University, Nekemte, Ethiopia; 4 Department of Nursing, College of Health Sciences, Diredawa University, Diredawa, Ethiopia; 1. IRCCS Neuromed 2. Doctors with Africa CUAMM, ITALY

## Abstract

**Background:**

First-line antiretroviral treatment failure has become a public health concern in high, low and middle-income countries with high mortality and morbidity In Ethiopia, around 710,000 peoples were living with HIV and 420,000 of them were receiving ART in 2017. Little is known about the magnitude of first-line ART treatment failure and its associated factors in Ethiopia, particularly in the study area. Therefore, this study was aimed to find the magnitude of first-line ART treatment failure and its associated factors among adult patients attending ART clinic at Southwest shoa zone public hospitals.

**Methods:**

Institutions based cross-sectional study was employed from February 1 to April 2, 2019. An interviewer administered questionnaire was used to collect data from 350 adult patients on ART using a systematic random sampling technique. The collected data were coded and entered into Epidata version 3 and exported to STATA SE version 14 for analysis. Bivariable and multivariable logistic regression was done to identify factors associated with first-line ART treatment failure. At 95% confidence level strength of association was measured using Odds ratio. Variables with a p-value of ≤ 0.25 in the bivariable analysis were considered as a candidate variable for multivariable analysis. To get the final variables step-wise backward selection procedure was used and those in the final model were selected at a p-value <0.05. Finally, texts, simple frequency tables, and figures were used to present the findings.

**Results:**

In this study the magnitude of first-line ART treatment failure was 33.42%. Absence of baseline opportunistic infection AOR = 0.362 (95%CI0.178, 0.735), Staying on first-line ART for <5 years AOR = 0.47 (95%CI 0.252, 0.878), Nevirapine containing ART regimen AOR = 3.07 (95%CI 1.677, 5.63), Baseline CD4 count ≥100 cells/mm3 AOR = 0.299 (95%CI 0.152 0.591), absence of opportunistic infections after ART initiation AOR = 0.257 (95%CI 0.142, .467), time taking greater than an one-hour to reach health facility AOR 1.85 (95%CI 1.022 3.367) were significantly associated with first-line ART treatment failure.

**Conclusion:**

The magnitude of first-line ART treatment failure was high in the study area. Base-line opportunistic infection, duration on first-line ART, NVP based ART, Baseline CD4 count level, OI after ART initiation, and time it takes to reach health facility were independent determinants of first-line ART treatment failure.

## Introduction

The Joint United Nations Program on HIV/AIDS (UNAIDS) estimates that around 36.9 million peoples were living with HIV worldwide in 2017 among which 21.7 million of them have access to antiretroviral therapy [1]. The African region also accounts for over two- thirds of the global HIV infections as 25.7 million peoples in Africa were living with HIV at the end of 2017 [2].

The number of adult peoples infected with Human Immunodeficiency Virus (HIV) in Ethiopia was estimated as 678,165 in 2018, with a prevalence of 1.15% and 9,294 annual death related to AIDS. In Oromia regional state of Ethiopia, about 165,911 peoples were living with HIV infection, and 2,230 of adult patients were died due to AIDS in 2018 [3]. The only option to sustain the life of the patient living with HIV is compliance to ART treatment regimens. Even though Antiretroviral therapy (ART) for HIV infection was started in 1987 [4], it was started in 2003 as a fee-based and 2005 as a free government service in Ethiopia [5].

In addition to increased morbidity and mortality associated with HIV/AIDS, ART treatment failure has become a public health concern in low and middle-income countries. There are three diagnostic methods of ART treatment failure. These are; virological, immunological and clinical failure. Virological failure is diagnosed when the plasma viral load is more than 1000 copies/ml in two consecutive measurements within a three months interval despite adherence to the ART treatment regimen. Whereas, immunological failure is defined as a CD4 count of ≤ 250 cells/ mm3 following clinical failure or persistent CD4 cells/mm3 levels below 100 cells /mm3. Clinical failure happens when new or recurrent WHO stage 4 or some stage 3 manifestations happen after six months of ART initiation [6].

Antiretroviral therapy lowers the viral load and the risk of opportunistic infections that decreases the rate of death from HIV/ Acquired immunodeficiency syndrome (AIDS). However, treatment failure has become a common significant risk of death from AIDS [7]. Findings from systemic review and meta-analysis conducted in Sub- Saharan Africa (SSA) indicated first-line ART treatment failure is 16% [8]. Evidence indicated that about 15.3% of people living with HIV/AIDS who were on first-line ART treatment-experienced first-line ART treatment failure [9]. In the northern part of Ethiopia ART treatment failure account 20.3%, 13.2%, and 14.7% with clinical, immunological, and virological failure respectively [10]. As a review from sixteen sub- Saharan Africa indicated 1·6 peoples per 100 on first-line ART shifted to second-line ART in each year [11]. By 2030 the numbers of patients on second-line ART will be increased to 0·8–4·6 million (6·6%– 19·6%) while the range of patients on first-line ART will remain constant [12]. Some research findings indicated that duration on ART in greater than six years, Cd4 < = 199 are some factors contributing to first-line ART treatment failure [10].

The first-line ART regimen is the best for virologic suppression and early immune recovery and prevents the occurrence of clinical failure than second-line ART [13]. If patients shifted from first-line ART to second-line ART, drug resistance will be increased, the cost of second-line ART is very high and it is more toxic and not convenient for patients, the chance of viral repression is low and the death rate is high for patients taking second-line ART treatment [14, 15].

Even though existing pieces of evidence showed CD4 < = 199 cells/mm3, taking ART for greater than six years and opportunistic infections as associated factors with first-line ART treatment failure, magnitude, and factors associated with first-line ART treatment failure is not adequately studied yet in the study area. Therefore, this study aimed to assess the magnitude of first-line ART treatment failure and its associated factors among adult patients attending ART clinics of southwest shoa zone public hospitals. The finding of this study is helpful to health care providers and health care planners to design effective strategies to prevent first-line ART treatment failure. Furthermore, it is useful to patients themselves in sustaining their life and improving their survival status.

## Methods

### Study design and setting

A Facility-based cross-sectional study was employed among HIV-infected adult patients on ART at Tulubolo general hospital and St. Luke referral hospital from February 1 to April 2, 2019. St. Luke Referral hospital is located in Oromia Regional state at 114 km southwest of Addis Ababa the capital city of Ethiopia. St. Luke's referral hospital is found in a town of the zone, Woliso. In St. Luke referral hospital ART clinic started in 2006 and it currently giving ART services for a total of adult 1433 HIV positive patients. Now, it is serving a population in the catchment area of 1.3 Million people. This hospital has 200 beds and the total peoples receiving ART at this hospital is 1563 adults and children. Again Tulubolo general hospital is located southwest from Addis Ababa the capital city of Ethiopia. It is located 90km on the road in the direction of the southwest from Addis Ababa and begun functioning in the year 2010. Its ART clinic is currently serving 201 patients from which 174 are adults while 27 are patients’ age less than fifteen years old who were treated in the pediatric out-patient department.

### Source and study population

The source population was all patients taking ART at both hospitals while study populations were adult patients who were taking ART during the study period and who were aged ≥15years old and who were on first-line ART for greater than 6 months during the data collection period. Patients on ART who were involuntary to participate; have incomplete data and transferred in patients during being on second-line ART from other health institutions were excluded from the study.

### Sample size and sampling procedure

**Fig 1:**
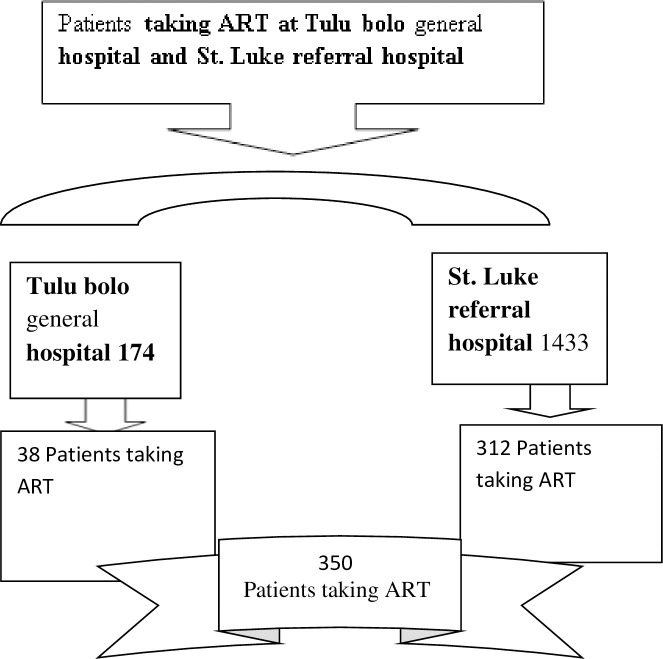
Schematic presentation of proportional allocation of patients taking ART at public health facilities in South West Shoa Zone, 2019.

To get the required sample size, known significantly associated variables such as the duration on first-line ART for greater than six years, having a history of loss to follow up, having baseline hemoglobin <11g/dl, CD4 cell count ≤200 cell/mm and BMI <18.5g/dl were used. Epi Info version 7 was used to compute the required sample size by considering the following parameters; 5% margin of error, 80% power, and 36.5% expected frequency. Hemoglobin <11g/dl yields the largest sample size292 and adding a 20% non-response rate yields the final sample size of 350. This sample was proportionally allocated to the two hospitals as per their patient flow to ART clinics during the study period (Fig 1).

A systematic sampling technique was used to get the individual participants. To reach the individual to be involved in the study first the schedule of the patients visit the hospital within the study period was identified from the flow chart of the patient in those hospitals. With this regard, from St. Luke's referral hospital 560 patients on ART were appointed to visit the hospital in the study period to take ART medication. The sampling interval for each hospital was approximately two and the lottery method was used to select the first patient to be interviewed. To get 312 participants at St. Luke referral hospital, starting from the second patient visited ART clinic every second patient coming to the ART clinic was interviewed. Similarly in Tulu bolo general hospital, 75 patients were appointed to visit the hospital in the study period. Therefore, to get 38 patients every second patient coming to the ART clinic starting from the first patient was selected.

### Data collection tool and procedure

The study tool was developed from different relevant pieces of literature [10, 16], and part of it was from ART guideline of the federal ministry of health of Ethiopia, ART follows up form, case manager sheets and patients’ medical cards [5], The question has five parts like Socio-demographic characteristics, laboratory measures, ART treatment-related conditions, clinical characteristics of patients, health facilities related questions and behaviors of HIV/ AIDS patients those who were on ART. The data were collected by three BSc nurses two at St. Luke referral hospital while the remaining one at Tulubolo hospital. The supervisor was monitoring the data collection process at each hospital daily. Data were extracted from patients’ charts/record reviews/ and the patient himself /herself by using a pretested structured checklist. When the incomplete card or involuntary patient appeared, the next patient was selected and interviewed. Regarding adherence status good adherence (> 90) was recorded if the patient miss to take ≤2 of 30 prescribed doses or ≤3 doses of 60 prescribed dose, fair adherence (85–94%) if the patient miss to take 3–5 of 30 prescribed doses or 3–9 doses of 60 prescribed dose and poor Adherence (<85%) if the patient miss to take less 6 of 30 prescribed doses or >9 doses of 60 prescribed dose [5]. All patients with age greater than fifteen years old were participated in this study. Patients taking ART in less than six months were excluded because we can’t confirm ART treatment failure within six months of ART treatment failure.

In this research all criteria for ART treatment failure confirmation was used (That is all virological, immunological, or clinical failure). Virological failure is diagnosed when the plasma viral load is more than 1000 copies/ml in two consecutive measurements within a three months interval despite adherence to the ART treatment regimen. Whereas, immunological failure is defined as a CD4 count ≤ 250 cells/ mm3 following clinical failure or persistent CD4 cells/mm3 levels below 100 cells /mm3. Clinical failure happens when new or recurrent WHO stage 4 or some stage 3 manifestations happen after six months of ART initiation initiation [6].

### Data quality assurance and analysis

Initially, the tool was prepared in the English language and translated to the local language, Afan Oromo, and then retranslated back to the English language by language experts to check the consistency. A two-days training was given for both data collectors and supervisors on confidentiality and privacy of data and how to collect the information from patients and the card. The tool was pretested on 5% of sample size at Amaya hospital and a necessary amendment was made. The data collection process was supervised and completeness was checked daily by the supervisor and principal investigator. Epi data version3 was used for data entry and exported to STATA SE version 14 for analysis. Firs descriptive statistics were computed. Media with Standard Deviation (SD) was used for a continuous variable like age.

Bivariable and multivariable logistic regression was used to identify factors associated with first-line ART treatment failure. Variables with a p-value of ≤0.25 in the bivariable analysis were considered as a candidate variable for multivariable analysis. To get the final variables step-wise backward selection procedure was used and those in the final model were selected. Log-likelihood goodness of fit test model was used. With 95% CI Crude and adjusted odds ratio was computed and statistical significance was declared at a P-value of <0.05. Finally, the final finding was presented by description, simple frequency tables, and graphs.

### Ethics approval and consent to participation

To for this research Ethical clearance was obtained from the Ethical review committee of Debre Markos University, college of health sciences in Ethiopia. After formal letter is written to study area data collection was started and verbal consent was obtained from participants. The supervisor was monitoring the data collection process at each hospital daily and We obtained verbal consent from respondents that was ticked on questionnaire sheet with agree for those willing to participate or disagree for those not willing to participate in the study. We encountered only two participants aged 15 years for which we obtained the consent from their family.

## Result and discussion

### Socio-demographic characteristics of the participants

In this study, a total of 350 patients who were taking ART at both hospitals were involved yielding a response rate of 100%. The median age of the participants was 45years (IQR 24, 44). Regarding educational status, around one-fourth of the participants were can’t read and write 87 (24.86%). Less than a quarter of the participants have no someone to support them 55 (15.71%). More than half of them were a follower of orthodox Christianity 222 (63.43%) (Table 1).

**Table 1:** Socio-demographic characteristics of patients taking ART drugs at ART at public health facilities in South West Shoa Zone, 2019.

Characteristics	Options	Frequency	Percent (%)
Sex	Male	166	47.43
	Female	184	52.57
Age	< = 35 years	221	63.14
	>35 years	129	36.86
Ethnicity	Oromo	258	73.71
	Amara	61	17.43
	Guraghe	27	7.71
	Others	4	1.14
Marital status	Single	40	11.43
	Married	189	54.00
	Separated/divorced	56	16.00
	Widowed	65	18.57
Education status of the patients	Can’t read and write	87	24.86
	Primary	156	44.57
	Secondary	81	23.14
	College and above	26	7.43
Occupation	Farmer	100	28.57
	Employed	45	12.86
	Students	21	6.00
	Merchant	116	33.14
	Laborer	44	12.57
	Others	24	24
Religion	Orthodox	222	63.43
	Protestant	101	28.86
	Muslim	27	7.71
Support	Yes	295	84.29
	No	55	15.71

(Other ethnicity Guraghe, Tigray walayita, Kebene. Other occupation, driver, carpenter, tailor, police)

### Clinical characteristics of the participants

**Fig 2:**
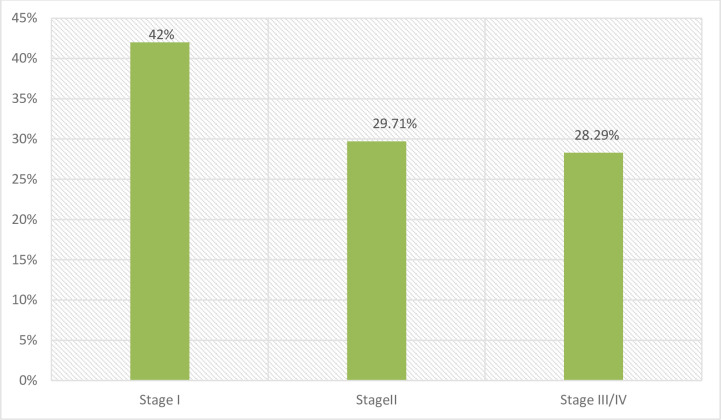
Baseline WHO stage of HIV/AIDS of patients taking ART drugs at ART at public health facilities in South West Shoa Zone, 2019.

Concerning to baseline WHO stage of HIV, relatively nearly half of them were in stage one during initiation of ART 147 (42.00) (Fig 2).

Regarding baseline functional status greater than three fourth of the participants were categorized in working state 289 (82.57%). Concerning baseline body mass index, greater than half of the participants were having BMI >18.5 kg/m2 209 (59.71%) while less than a quarter of them were having BMI ≤16kg/m2 19 (5.43%). Most of the participants have no history of chronic non-communicable diseases during taking ART 309 (88.29%) (Table 2).

**Table 2:** Clinical characteristics of patients taking ART drugs at public health facilities in South West Shoa Zone, 2019.

Characteristics	Options	Frequency	Percent (%)
Base line functional status	Working	289	82.57
	Ambulatory and bed ridden	61	17.43
Base line body mass index	< = 16	19	5.43
	16.01–18.5	122	122
	>18.5	209	59.71
Base line opportunistic infection	Yes	230	65.71
	No	120	34.29
OI after initiate ART	Yes	136	38.86
	No	214	61.14
Chronic non communicable diseases during on first line ART	Yes	41	11.71
	No	309	88.29
Presence of malnutrition during on first line ART	Yes	45	12.86
	No	305	87.14
Cotrimoxazole taken or not	Yes	299	85.43
	No	51	14.57

### ART treatment related conditions of the participants

**Fig 3:**
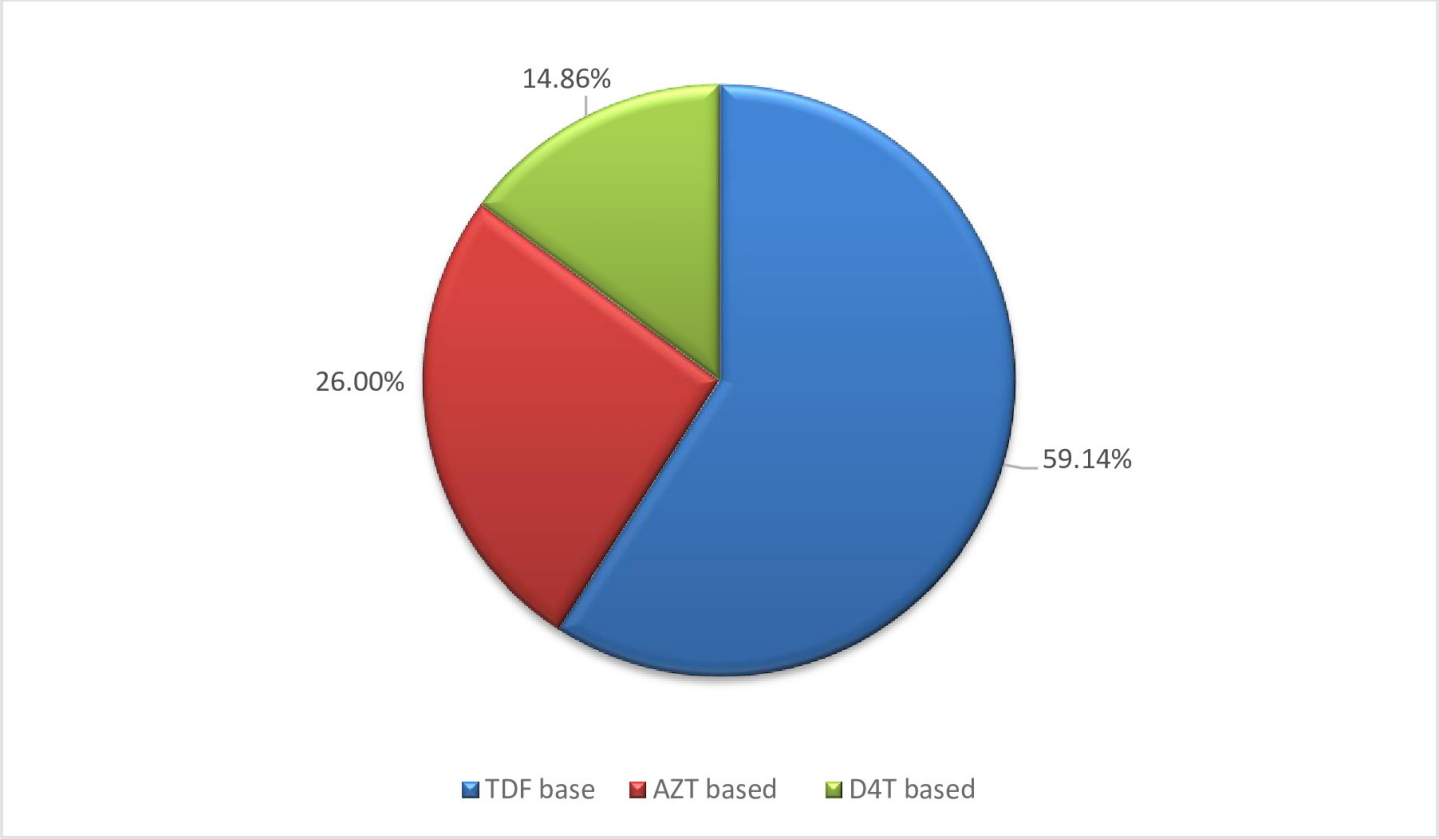
Base line NRTI based art of patients taking ART drugs at ART at public health facilities in South West Shoa Zone, 2019.

Regarding the NRTI based ART regimen, greater than half of the patients received TDF containing ART regimen 207 (59.14%) (Fig 3).

Concerning to NNRTI based ART regimen, greater than half of the participants received EFV containing ART regimen 210 (60.00%). Only less than a quarter of the participants had a history of loss to follow up 42 (12.00%). Greater than three fourth of the participants have good ART adherence status 275 (78.57%). Most of the participants have no history of drug side effects 329 (94. 00%). Greater than half of the participants have stayed on first-line ART for less than five years 199 (56.86%) (Table 3).

**Table 3:** ART treatment related condition of patients taking ART drugs at public health facilities in South West Shoa Zone, 2019.

Characteristics	Options	Frequency	Percent (%)
NRTI based	TDF based	207	59.14
	AZT based	91	26.00
	D4T based	52	14.86
NNRTI Based	EFV based	210	60.00
	NVP based	140	40.00
Lost to follow up	Yes	42	12.00
	No	308	88.00
ART adherence status	Good	275	78.57
	Fair	43	12.29
	Poor	32	9.14
Disclosure being HIV positive to family/others	Yes	269	76.86
	No	81	23.14
occurrence of drug side effect	Yes	21	6.00
	No	329	94.00
Duration on first line ART	< = 5yrs	199	56.86
	>5yrs	151	43.14
First line ART drug substitution	Yes	92	26.29
	No	258	73.71
Time lag to start ART after diagnosed positive	The same day	51	14.57
	1 to 24 month	273	78.00
	Greater than 24 month	26	7.43

### Health care facility, laboratory result and patients related conditions of the participants

With regard to the time it takes to reach the health facility, around three fourth of respondents reported an hour 246 (70.29%). Most participants had begun ART at hospitals 331 (94.57%). Almost all of the participants have no history of taking Post-exposure prophylaxis 341 (97.43%) Regarding—baseline laboratory findings around three-fourth of the participants have baseline Cd4 greater than 100 counts 272 (77.71%) and most of them have base-line hemoglobin greater than 10mg/dl (Table 4)

**Table 4:** Health care facility, laboratory result and patient related condition of patients taking ART drugs at ART at public health facilities in South West Shoa Zone, 2019.

Characteristics	Options	Frequency	Percent (%)
Treatment failed confirmed	Yes	117	33.42
	No	233	66.58
Time it ask to go health facilities	< = 1 hr	141	40.28
	>1hrs	209	59.72
Health facility where start first line ART	Hospitals	331	94.57
	Health center	19	5.43
Suspected route of infection	I don’t know	66	18.86
	Sexual intercourse	264	75.43
	Sharp material	20	5.71
Mode of first identified ART infection	VCT	76	21.71
	PICT	274	78.29
History taking of Post exposure prophylaxis	yes	9	2.57
	No	341	97.43
Baseline CD4 count	<100	78	22.29
	> = 100	272	77.71
Baseline hemoglobin	<10	17	4.86
	> = 10	333	95.14

### Analysis of determinants of first line ART treatment failure

Among the candidate variables for Multivariable logistic regression, only nine of them remained in the final regression model. Accordingly, variables as baseline opportunistic infections, presences of opportunistic infection after ART initiation, NNRTI based first-line ART, adherence status, base-line Cd4 count, durations on first-line ART and time it takes to reach health facilities remained in the multivariable analysis model.

A Finding from the multivariable analysis indicated that there was a positive association between first-line ART treatment failure and having base-line opportunistic infections. That those who have no OI during ART initiation were 64% times less likely faced first-line ART treatment failure than their counter-parts (AOR = 0.362 (95%CI 0.178, .735). Also, the result indicated that those patients who have stayed on first-line ART for less than five years were 53% times less likely experienced the occurrence of first-line ART treatment failure than those who have stayed on first-line ART for greater than five years AOR = 0.47 (95%CI 0.252, 0.878).

Regarding baseline CD4 count, those who have started ART with Cd4 greater than 100 have 70% less likely faced the occurrence of first-line ART treatment failure AOR = 0.229 (95% CI .152 .591). In addition, the finding showed that those who have started ART at health center 3.56 times more likely experience first-line ART treatment failure than those who have started at the hospitals AOR = 3.47 (95%CI 1.08,11.736). Furthermore, the finding indicated that the presence of OI after ART initiation was associated with first-line ART treatment failure. Those who reported no opportunistic infections after ART initiation had 74% times lower likelihood of developing first-line ART treatment failure than their counterparts AOR = 0.257 (95%CI 0.142,0.467).

Moreover, the finding also indicated that patients who have started ART regimen containing NVP have a 3.07 times higher likelihood of developing first-line ART treatment failure as compared to those who received EFV containing ART regimen AOR = 3.07 (95%CI 1.677,5.63). With regard to time to reach a health facility, those patients who were wasting more than an hour were 1.85 times more likely to develop first-line ART failure as compared to patients wasting less than an hour to reach the health facility. AOR = 1.85 (95%CI1.022 3.367) (Table 5).

**Table 5:** Multivariable analysis of predictors of first line ART treatment failure.

Characteristics		First line ART treatment failure		COR (95%CI)	AOR (95%CI)	P-value
		Yes (%)	No (%%)			
**Base line opportunistic infection**	Yes	99 (28.28)	131 (37.43)	1		
	No	18 (5.14)	102 (29.15)	0.233 (.132, .410)	0.362 (.178, .735)	0.005*
**Duration on first line ART**	< = 5yrs	63 (18)	136 (38.85)	0.832 (.532, 1.301)	0.47 (.252, .878)	0.018*
	>5yrs	54 (15.42)	97 (27.71)	1		
**Baseline CD4 count**	<100	46 (13.14)	32 (9.12)	1		
	> = 100	71 (20.28)	201 (57.42)	0.245 (.145, .415)	0.299 (.152, .591)	0.001*
**Art adherence status**	Good	72 (20.57)	203 (58)	1		
	Fair	21 (6)	22 (6.28)	2.691 (1.397, 5.184)	1.754 (.791, 7.38)	0.166
	Poor	24 (6.85)	8 (2.28)	8.458 (3.636, 19.67)	2.27 (0.823, 6.27)	0.113
**Health facility where start first line ART**	Hospitals	105 (30)	226 (64.57)	1		
	Health center	12 (3.42)	7 (2)	3.689 (1.412, 9.641)	3.56 (1.08, 11.736)	0.037 *
**OI after initiate ART**	Yes	79 (22.57)	57 (16.29)	1		
	No	38 (10.86)	176 (50.28)	0.155 (.095, .253)	0.257 (.142, .467)	<0.001*
**NNRTI Based ART**	EFV based	49 (14)	161 (46)	1		
	NVP based	68 (19.42)	72 (20.57)	3.103 (1.957, 4.918)	3.07 (1.677, 5.63)	<0.001*
**Time it ask to go health facilities**	< = 1 hr	38 (10.85)	103 (29.42)	1		
	>1hrs	79 (22.57)	130 (3)	1.647 (1.034, 2.623)	1.85 (1.022, 3.367)	0.042*

## Discussion

Even though the accessibility of ART medication is high, first-line ART treatment failure has become a public health agenda on the top of HIV/AIDS morbidity and mortality in low and middle-income countries. There is inadequate information on the magnitude of First-line ART treatment failure and its associated factors among adult patients in Ethiopia, particularly in the study area. Thus, this study aimed at determining the magnitude of First-line ART treatment failure and its associated factors among adult patients attending ART clinics in selected hospitals of the Southwest shoa zone in Ethiopia.

This study pointed out that the overall First-line ART treatment failure is 33.42% (95%CI 20.12, 42) in the study area. This finding is higher than the studies done in India (7.69%), a meta-analysis done in Ethiopia (15.9%) [17, 18]. The discrepancy in this magnitude might be due to the differences in living conditions of the population, sample size difference, difference in study design and year of the study.

Regarding factors associated with first line ART treatment failure in the present study, patients who had no opportunistic infections had a 64% lower likelihood of developing First-line ART treatment failure as compared to their counterparts. This finding is supported by evidence from India, Western Oromia, Arbaminch, and Ethiopian meta-analysis [17–20]. Not only had the base-line opportunistic infections the finding also showed that the occurrence of opportunistic infection after ART initiation is associated with first line ART Treatment failure. This is because patients who have no opportunistic infections have relatively strong immunity and less viral load that help them to withstand treatment failure. Again patients with other opportunistic infection have double burden and due to this ART treatment failure is high among them than those who have no opportunistic infection. Patients who have opportunistic infection may take other drugs that interact with ART that may again lead drug-drug interaction and brings ART treatment failure. In addition, the study found that a baseline CD4 count of greater than 100cells/mm3 is a protective factor for first-line ART treatment failure. For this finding similar existing evidences upkeep this finding [17, 20–22]. This suggests the higher CD4 count, the lower viral load which protects against treatment failure. Higher CD4 level is guarantee to prevent opportunistic infection and decrease viral load.

Regarding NNRTI, nevirapine based first-line ART regimen has a higher probability of treatment failure than Efavirenz based regimen of NNRTI. This finding is supported by a study conducted in Tanzania [23]. The reasons behind this may be NVP is more difficult for the patients to manage its side effect as abdominal pains, vomiting, rash, and others which are more common for NVP than for EFV. Again since NVP is taken twice a day that leads disturbance in adherence status that may synergically contribute to treatment failure.

Times it takes to go to a health facility is also associated with first-line ART treatment failure, that the time required to reach health facility increase the chance of treatment failure also increased. This may be due to the patients are bothered to go to the health facility due to distance and sometimes they may refuse to go there due to many reasons as tiredness, lack of transport and other un-conducive environment.

Again, regarding where to initiate ART, the finding revealed that starting ART at health center was associated with first-line ART treatment failure. This may be due to differences in knowledge gab of the participants since most of those who have started in the health center were from rural areas. In addition, patients are relatively better counselled in hospitals than in health center as in Ethiopia HIV positive patients are linked to counseling services in hospitals where as no counseling services in health centers. In Ethiopia there is casa manager at Hospital but not at health center. Case manager is HIV positive patients who employed in the hospital to give counseling service for the HIV positive patients. So giving counseling service as hospital is greater in hospital than in health center. So this low counseling service at health center may lead First-line ART treatment failure.

This study finding again revealed that as the time to stay on first-line ART increases, the occurrence of first-line ART failures was increased too. This finding is supported by a study conducted in Tanzania [22]. This is because longer duration on first-line ART increases chance of viral mutation, resistance of the virus, the chance of the individuals to develop more opportunistic infection, and decreases immunity of the patients which in turn lead to conducive environments for the viral replications and eventually ART treatment failure.

Even though previous study findings revealed that adherence to first-line ART regimen is a protective factor to first-line ART treatment failure [18, 21, 24, 25], the current study found no statistically significant association between first-line ART adherence and first-line ART treatment failure. This might be due to variation in sample size or research design.

### Limitation of the study

This study is cross-sectional study and more strong study designs with large sample size is recommended to identify magnitude and factors associated with first line ART treatment failure.

## Conclusion

The overall first-line ART treatment failure among adult patients is surprisingly high in the study area. Opportunistic infection, Duration of first-line ART, Baseline CD4 count, Health facility where first-line ART started, Opportunistic infection after ART initiated, and Nonreversible transcriptase inhibitors based ART regimen were independent determinants of First-line ART treatment failure. Therefore, all responsible bodies should pay attention to minimize or alleviate identified factors in order to reduce the risk of developing first-line ART treatment failure

## Supporting information

S1 Data set (DTA)

## Abbreviations

AOR: COR Crude Odd ratio
ART: Antiretroviral therapy
NNRTI: non- nucleotide reverse transcriptase inhibitors
